# Dual transcriptome sequencing reveals resistance of TLR4 ligand-activated bone marrow-derived macrophages to inflammation mediated by the BET inhibitor JQ1

**DOI:** 10.1038/srep16932

**Published:** 2015-11-19

**Authors:** Amitabh Das, Jin Choul Chai, Chul-su Yang, Young Seek Lee, Nando Dulal Das, Kyoung Hwa Jung, Young Gyu Chai

**Affiliations:** 1 Department of Bionanotechnology, Hanyang University, Seoul, 133-791, Republic of Korea; 2 Department of Molecular & Life Sciences, Hanyang University, Ansan, 426-791, Republic of Korea; 3 Epigenetics Drug Discovery Unit, Division of Structural & Synthetic Biology, RIKEN Center for Life Science Technologies, 1-7-22 Suehiro-cho, Yokohama 230-0045, Japan; 4 Institute of Natural Science & Technology, Hanyang University, Ansan, 426-791, Republic of Korea

## Abstract

Persistent macrophage activation is associated with the expression of various pro-inflammatory genes, cytokines and chemokines, which may initiate or amplify inflammatory disorders. A novel synthetic BET inhibitor, JQ1, was proven to exert immunosuppressive activities in macrophages. However, a genome-wide search for JQ1 molecular targets has not been undertaken. The present study aimed at evaluating the anti-inflammatory function and underlying genes that are targeted by JQ1 in LPS-stimulated primary bone marrow-derived macrophages (BMDMs) using global transcriptomic RNA sequencing and quantitative real-time PCR. Among the annotated genes, transcriptional sequencing of BMDMs that were treated with JQ1 revealed a selective effect on LPS-induced gene expression in which the induction of cytokines/chemokines, interferon-stimulated genes, and prominent (transcription factors) TFs was suppressed. Additionally, we found that JQ1 reduced the expression of previously unidentified genes that are important in inflammation. Importantly, these inflammatory genes were not affected by JQ1 treatment alone. Furthermore, we confirmed that JQ1 reduced cytokines/chemokines in the supernatants of LPS treated BMDMs. Moreover, the biological pathways and gene ontology of the differentially expressed genes were determined in the JQ1 treatment of BMDMs. These unprecedented results suggest that the BET inhibitor JQ1 is a candidate for the prevention or therapeutic treatment of inflammatory disorders.

Macrophages are a major cell population of the innate immune system^1^. These cells play an important role in this system. Tissue resident macrophages, which can be derived from embryonic precursors, are seeded before birth and can sustain themselves in adults by self-renewal, which is characteristic of adult bone marrow-derived macrophages^2^,^3^. Macrophages are able to become activated in response to infection, inflammation or injury, and their activation is involved in the production of various pro-inflammatory mediators, such as reactive oxygen species (ROS), nitric oxide (NO) and prostaglandin E2 (PGE2), and a variety of pro-inflammatory cytokines, including interleukin Il1β, Il6 and tumour necrosis factor (Tnf-α)^4^,^5^. Although macrophage activation is considered a protective mechanism that is involved in pathogen infection clearance and in regulating tissue repair and recovery, excessive or persistent activation of these innate immune cells contributes to the pathogenesis of both metabolic and inflammatory disorders^6^. However, the protective mechanisms and the damaging macrophage phenotypes have not been fully elucidated. Considering the significant impact of macrophage-mediated innate immunity, preventing the harmful effects that are associated with their activation may offer new therapeutic approaches for the treatment of inflammatory disorders.

Macrophages express numerous pattern recognition receptors (PRRs) that detect and respond to the presence of various stimuli/toxins^7^,^8^. Among these, lipopolysaccharide (LPS), the Toll-like receptor 4 (TLR4) ligand, is one of the most potent stimuli for macrophage activation. LPS, or endotoxin, is a major outer membrane component of Gram-negative bacteria and induces intracellular signalling pathways, leading to the induction a broad gene expression program that constitutes the innate immune response to Gram-negative bacterial infections^9^. Previous studies demonstrated that abundant pro-inflammatory cytokines can result in excessive inflammation and tissue damage, which contributes to inflammatory disorder pathogenesis^10^. LPS can reprogram transcription through its ability to activate acetylation of the lysine residues that are present in histone tails, a general hallmark of gene activation^11^. These acetylated lysines are recognized by highly conserved chromatin readers, which are designated as N-terminal bromodomains. These domains are common in all four members of the bromodomain and extra terminal domain (BET) family of adaptor proteins (Brd2, Brd3, Brd4 and Brdt). In humans, at least 40 bromodomain proteins are present, including histone acetyltransferases, helicases, scaffolding proteins and other cofactors that control gene transcription^12^. These events raise the possibility that bromodomain proteins regulate acetylated, histone-packaged inflammatory gene expression programs that are associated with various human diseases.

Recently, James Bradner and colleagues discovered a potent and highly specific bromodomain inhibitor, JQ1, which displaces BET bromodomains from acetylated lysines on chromatin^12^. The inhibitor represses downstream gene expression by competitively binding to BET proteins and displacing BET proteins from acetylated lysines on chromatin. These proteins emerged as attractive therapeutic targets in the treatment of inflammation and cancer^12^,^13^. JQ1 was shown to control the expression of numerous genes involved in the cell cycle, cell growth, inflammation and cancer, which suggests that the products of these genes function as epigenetic signalling proteins that regulate transcription in a cell context-dependent manner^14^,^15^. These outcomes indicate the possibility of using JQ1 as a potential therapeutic target for modulating gene expression programs that are associated with a diverse range of pathologies, predominantly cancer and inflammatory diseases. These compounds were demonstrated to exhibit potent inhibitory activity against a range of cell lines that were derived from haematological malignancies, including multiple myeloma, acute myeloid leukaemia, Burkitt’s lymphoma, and mixed-lineage leukaemia (MLL)^12^,^16^,^17^. However, the targeting of BET protein functions by JQ1 in non-malignant cells remains largely unexplored^18^. Indeed, considering the significance of BET proteins in inflammation, it is important to evaluate the possibility that JQ1 may be exploited as a next-generation anti-inflammatory treatment.

Although JQ1 or I-BET reduces inflammatory gene production in LPS-stimulated macrophages^6^,^13^, a genome-wide search for JQ1 molecular targets in LPS-activated BMDMs has not yet been performed. We therefore performed gene array and comparative gene expression profiling analyses of BMDMs that were treated with LPS, JQ1 or LPS + JQ1 using RNA sequencing (RNA-seq), a precise technique that is increasingly being used to study gene expression, as it provides unbiased profiles and the ability to identify novel transcribed regions compared with microarrays and can be extremely accurate if a sufficient level of coverage is obtained^19^,^20^. Validation techniques, such as quantitative real-time RT-PCR (qRT-PCR)^21^, have corroborated the accuracy of RNA-seq. To the best of our knowledge, this is the first study to apply these approaches to assess the JQ1-mediated global gene expression changes in BMDMs cells using RNA-seq analysis.

Our results show that JQ1 is a potent modulator of macrophage activation. In particular, JQ1 treatment resulted in a significant down-regulation of key inflammatory genes in LPS-activated macrophage cells. Importantly, these inflammatory genes were not affected by JQ1 treatment alone. Overall, the results suggested that JQ1 might be an effective therapeutic target with possible research and clinical value. Taken together, these findings establish a role for BET proteins in mouse macrophage stimulation and justify further testing of BET protein-targeting genes in inflammatory disorders.

## Results

### Distinct gene signatures were identified during the inflammatory response according to RNA-seq analysis in BMDMs

To establish a high-resolution transcriptome in response to LPS stimulation, we treated BMDMs with LPS for 1 and 4 h before cDNA library preparation for RNA-seq experiments. The RNA-seq transcriptional analysis was performed using two independent samples (biological replicates) of each treatment. The data from all of the experiments (each group) were combined, and the genes whose expression levels significantly differed were identified. We used a 1% false discovery rate (FDR), *P* ≤ 0.01, and fold change ≥1.5 log_2_ for up- or down-regulation as the criteria for defining the differentially expressed genes. We chose these time points for transcriptional profiling, as these time points were also used in another study^14^ that investigated the general induction pattern of BMDM activation with LPS.

**Figure 1:**
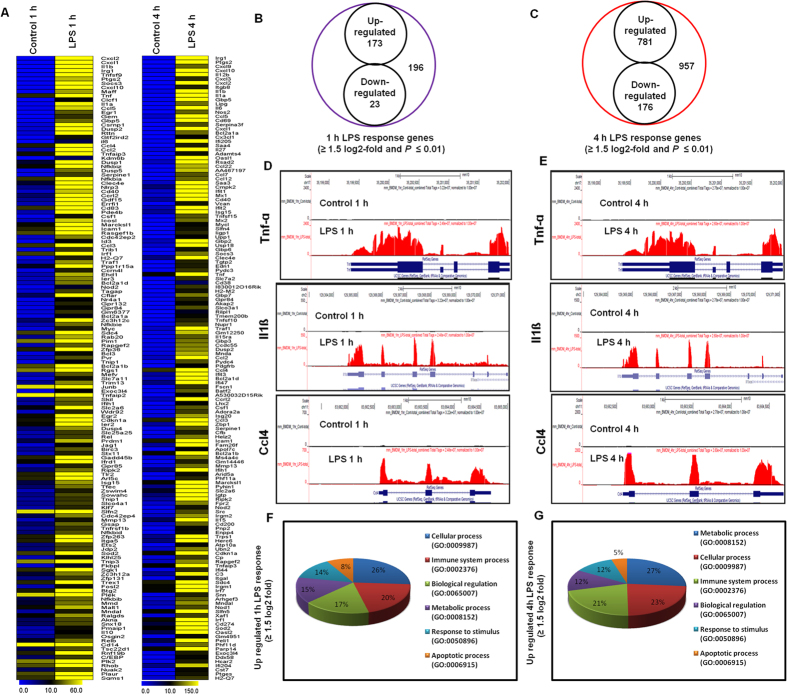
RNA-seq analysis reveals LPS-stimulated pro-inflammatory gene expression in BMDMs. (**A**) A heat map representing RNA-seq gene expression of the top 150 up-regulated (*P* ≤ 0.01, and fold change ≥1.5 log_2_) inflammatory genes in 1 h and 4 h LPS-stimulated BMDMs compared with the control. (**B**,**C**) A Venn diagram displaying the number of inducible or repressible (≥1.5 log_2_-fold) genes after 1-h and 4-h LPS-stimulated BMDMs. (**D**,**E**) UCSC Browser images representing the normalized RNA-seq read densities at 1 h and 4 h after LPS stimulation in the BMDMs compared with the controls. (**F**,**G**) Gene ontology analysis of the functional annotations that were associated with up-regulated genes at 1 and 4 h after LPS stimulation in the BMDMs.

The RNA-seq analysis revealed differentially expressed genes in the LPS-stimulated BMDMs at both time points. Specifically, 196 and 957 genes (increased and decreased in expression ≥1.5 log_2_-fold, respectively and *P* ≤ 0.01) were differentially regulated at 1 and 4 h, respectively. Among them, 173 and 781 genes were up-regulated, whereas 23 and 176 genes were down-regulated at 1 h and 4 h after LPS treatment, respectively (Fig. 1B,C). The following inflammatory response- and immune response-related genes exhibited the most dramatic induction levels following the LPS challenge: Nos2, interleukin and interleukin-related genes (Il12b, Il2rg, Il1β, Il1a, Il6, Il27, Il15ra, Il15, Il18, Il10ra, Il13ra1 and Il1rn); Tnf and Tnf-related genes (Tnf-α, Tnfaip3, Tnfaip2, Tnfsf9, Tnfrsf14, Tnip3, and Tnfrsf1b); a prostaglandin-related gene, Ptgs2; interferon-related genes (Ifi35, Ifi203, Ifi204, Ifi205, Oasl1, Oasl2, Oas1b, Oas1g, Oas2, Oas3, Mx1, Mx2, Ifit1, Ifit2, Ifit3, Ifi47, Isg15, Isg20, Zbp-1, Ifih1, and Ifi44); and cytokines or chemokines (Cxcl1, Cxcl2, Cxcl3, Cxcl9, Cxcl10, Cxcl16, Cx3cl1, Ccl5, Ccl22, Ccl4, Ccl7, Ccl2, Ccrl2 Ccl3, Ccl12, and Ccl9) (Fig. 1A,D,E)). We selected these genes based on biological processes and molecular gene ontology functions. As the down-regulated genes (Additional file 1: Supplementary Tables S1 and S2) were not associated with inflammation, only the up-regulated genes were further studied. We confirmed with gene ontology (GO) analysis (FDR 0.05) using the DAVID Bioinformatics Resources that the largest groups of LPS down-regulated transcripts were associated with regulation of metabolic process, cellular process, and localization in the BMDMs (Additional file 1: Supplementary Figure S1). We next performed functional classification analyses of the up-regulated genes (≥1.5 log_2_-fold and *P* ≤ 0.01) using the DAVID Informatics Resources through classification into GO categories (FDR 0.05) based on biological process (BP) and molecular function (MF) categories as well as KEGG (Kyoto Encyclopedia of Genes and Genomes) pathways. The genes that were up-regulated in response to LPS stimulation were involved in several BPs and MFs. We observed that the largest groups of genes were involved in immune system regulation and stimulus responses. Other pathways, such as cell death regulation, locomotion, and biological processes, were also identified in the differentially expressed gene analysis at 1 and 4 h after LPS stimulation in the BMDMs (Fig. 1F,G).

### Differential expression of multiple TF families in the LPS-stimulated BMDM RNA-seq data

**Figure 2:**
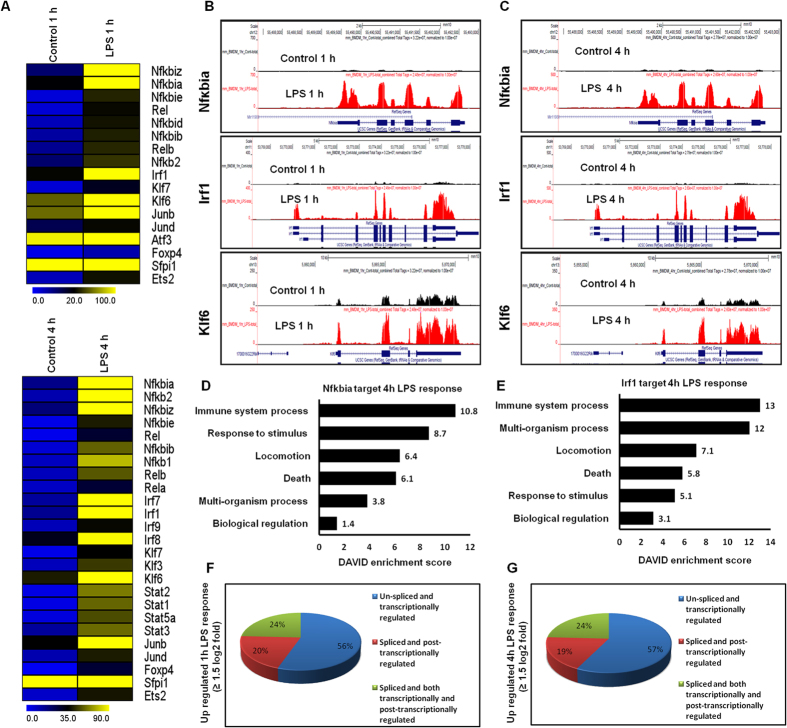
Transcriptomic analysis of the selected TF families in BMDMs. (**A**) A heat map represents differential expression of NFκB, Irf, Stat, Klf, and other TF gene families at 1 and 4 h after LPS stimulation in BMDMs. (**B**,**C**) UCSC Browser images representing normalized RNA-seq read densities for TF expression at 1 and 4 h after LPS stimulation in BMDMs compared with controls. (**D**,**E**) Results of the GO term analysis using DAVID on genes that were regulated by Nfκbia and Irf1 in 4 h LPS response BMDMs. (**F**,**G**) Transcriptional and post-transcriptional regulatory effects on the overall transcript output (*P* ≤ 0.01, and fold change ≥1.5 log_2_) in 1 and 4 h LPS-stimulated BMDMs.

Multiple TF families were identified among the differentially expressed genes (DEGs) that were significantly up-regulated at 1 and 4 h after LPS stimulation in the BMDMs (Fig. 2A). These TFs, including Irf, Kruppel-like factor (klf), NF-κB and signal transducer and activator of transcription (Stat), are important in inflammatory diseases^22^,^23^. The following TF families exhibited the most dramatic induction levels following the LPS challenge: the NF-κB-group of TFs (Nfκbia, Nfκb2, Nfκbie, Nfκb1, Nfκbib, Nfκbid, Nfκbiz, Rela, and Relb); the interferon-group of TFs (Irf1, Irf7, Irf8 and Irf9); klf-related TFs (Klf3, Klf6, Klf7); and the Stat-group of TFs (Stat1, Stat2, Stat3, and Stat5a). Interestingly, Irf2, Irf3, Irf4, Irf5, Irf6, Stat6, Klf1, Klf2, Klf4, and Klf5 were marginally expressed or were unaffected after LPS treatment, suggesting that LPS-induced gene expression is highly selective in BMDMs (Fig. 2A–C). More importantly, we observed that the Irf TFs (Irf1, Irf7, and Irf9) were not expressed in a macrophage cell line (RAW 264.7) in response to LPS, suggesting that Irf1, Irf7, Irf8 and Irf9 might be important regulators in the selective inflammatory gene expression that occurs in primary macrophages. However, we could not significantly distinguish other TF groups, such as NF-kB and Klf7, and proinflammatory gene expression programs between the primary macrophages and macrophage cell lines^24^. Additionally, the RNA-seq reads also revealed that Junb, Jund, Foxp4 and Ets2 were particularly up-regulated in the LPS-stimulated BMDMs (Fig. 2A). Next, we conducted IPA software to identify the target genes that were directly or indirectly activated by the identified TFs in response to LPS stimulation. Importantly, we found that the expression changes of the majority of the observed cytokines and chemokines were directly regulated by the identified TFs, including Stat1, Nfκbia, and Irf1 (Additional file 2). To further functionally classify the Nfκbia and Irf1-regulated genes, the data were functionally annotated using the DAVID 6.7 software package. Interestingly, we observed strong enrichments of GO terms for the Nfκbia, and Irf1-regulated transcripts that were associated with immune system processes, multicellular organism processes, locomotion and response to stimulus in LPS response BMDMs (Figs. 2D,E). Taken together, these findings specify that multiple TF families might be involved in the regulation of BMDM activation.

### Transcriptional and post-transcriptional regulation of LPS-stimulated BMDMs

Differentially expressed isoforms with different TSSs are transcriptionally regulated, while differentially expressed isoforms with the same TSSs are post-transcriptionally regulated^25^. In the present study, the transcripts, isoforms and TSSs of the up-regulated genes (≥1.5 log_2_-fold and *P* ≤ 0.01) in the LPS-stimulated BMDMs after 1 and 4 h were investigated. We defined three gene groups (Figs. 2F,G): A. Genes with one TSS and one isoform, which were classified as “un-spliced and transcriptionally regulated”; B. Genes with one TSS and more than one isoform, which were classified as “spliced and post-transcriptionally regulated”; and C. Genes with more than one TSS and more than one isoform, which were classified as “spliced and both transcriptionally and post-transcriptionally regulated”. These results revealed that differentially expressed isoforms with different TSSs offer an additional perspective of gene regulation in LPS-stimulated BMDMs.

### The BET inhibitor JQ1 reduces inflammatory responses in BMDMs

**Figure 3:**
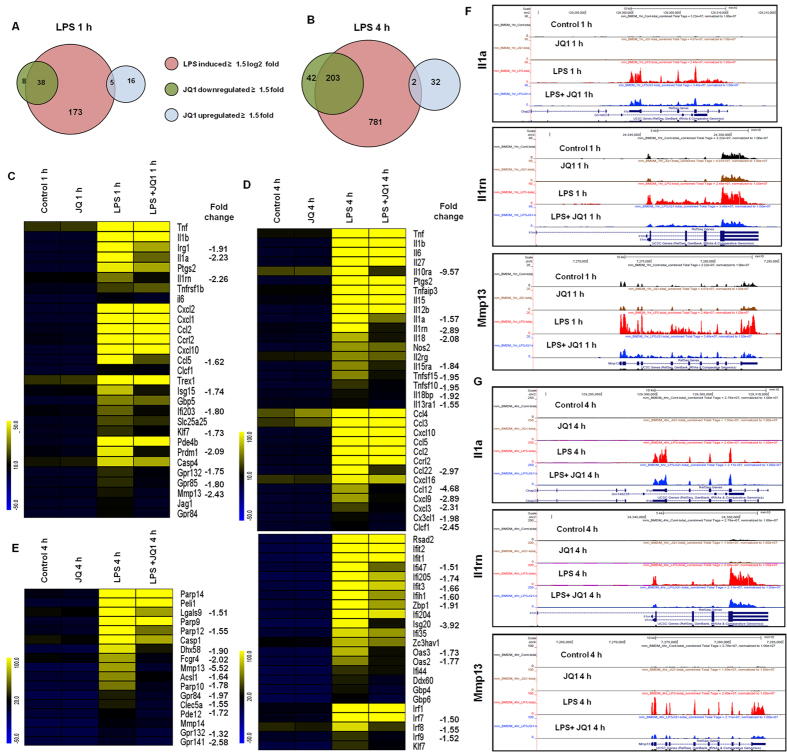
JQ1 suppresses a specific subset of LPS-inducible genes. (**A**,**B**) A Venn diagram displaying the number of LPS-inducible genes that were suppressed or up-regulated with JQ1 treatment at 1 or 4 h after LPS stimulation (left and right panels) of the BMDMs. (**C**–**E**) A heat map representation of gene expression levels that were selectively down-regulated (*P* ≤ 0.01, and fold change ≥1.5) by JQ1 at 1 and 4 h after LPS stimulation, from two independent BMDM experiments. Biological replicates (n = 2) for each condition were combined separately, and the heat maps were generated with the Multi Experiment Viewer (version 4.8) software. (F and G) UCSC Browser images representing the normalized RNA-seq read density in JQ1-down-regulated inflammatory genes at 1 and 4 h in the LPS-stimulated BMDMs compared with controls.

To investigate whether the broad-spectrum BET protein inhibitor JQ1 is also a broad-spectrum, anti-inflammatory agent, we tested its efficacy as an immunomodulatory drug that could counter macrophage-mediated inflammation. We exposed BMDMs to LPS and concomitantly treated them with JQ1 for both time periods and compared the transcriptomic profile from the group treated with LPS alone with that obtained from the group treated with LPS+JQ1. Many of the inflammation-related genes were significantly suppressed by JQ1. Indeed, we observed that 1 μM JQ1, a dose used in previous publications^26^,^27^, led to a marked reduction of inflammatory gene expression levels in the BMDMs. Treatment of BMDMs with JQ1 and LPS resulted in the down-regulation (*P* ≤ 0.01, and fold change ≥1.5) of 38 and 203 of the LPS-inducible genes at 1 and 4 h, respectively (Figs. 3A,B). JQ1 suppressed the expression of key LPS-inducible inflammation- and immunity-related genes, including Il1β, Il1a, Il6, Il15ra, Il18, Il18bp, Il10ra, Il13ra1 Il1rn, Irg1, Ccl2, Ccl5, Cxcl3, Cxcl9, Cx3cl1, Ccl12, Ccl22, TFs; Irf7, Irf8, Irf9, and klf7 (Fig. 3C–G). Interestingly, inhibition of the NF-κB group of TFs (Nfκbia, Nfκb2, Nfκbie, Nfκb1, Nfκbib, Nfκbid, Nfκbiz, Rela, and Relb) and the Stat group of TFs (Stat1, Stat2, Stat3, and Stat5a) were not suppressed by JQ1. A crucial inflammatory gene, Tnf-α (Additional file 1: Supplementary Figure S2), as well as other inflammation- and immunity-related genes, such as Ptgs2, Nos2, Ccl3, Ccl4, Cxcl1, and Cxcl2, were marginally affected or unaffected by JQ1, suggesting that the JQ1-treated, LPS-inducible gene expression profile is highly selective. Consistent with our findings, Nicodeme *et al.*^14^ reported that significant inflammatory genes, including Tnf-α and Ccl3, were unaffected by another synthetic BET family protein (I-BET) in BMDMs. They observed that following I-BET treatment, higher BET levels at the Tnf-α locus were associated with largely unchanged levels of positive transcriptional elongation factor b, RNA polymerase II, and RNA polymerase II S2. This is an important area that we are actively pursuing further.

### Effect of JQ1 alone on resting BMDM cells

We also evaluated the effect of JQ1 alone in resting BMDMs. The results showed that JQ1 alone, in the absence of LPS stimulation, also altered the expression of some genes, with a 1.5 log_2_-fold and *P* ≤ 0.01 cut-off value. Most of these genes do not have a well-established role in inflammation, whereas genes associated with inflammation (Ptgs2, Nos2, Il1β, Il1a, Il18, Il1rn, Tnf-α, Tnfaip3, Tnip3, Tnip1, Tnfaip2, Ifit1, Irf1, Irf7, Irf9, Cxcl10, Ccl4, Ccl7, Ccl2, Ccl3, Ccl12, and Ccl9) were unaffected or expressed insignificantly. A total of 21 and 34 genes (≥1.5 log_2_-fold and *P* ≤0.01) were up-regulated in the BMDMs that were treated with JQ1 alone at 1 and 4 h, respectively (Fig. 3A,B). Notably, we observed that chromatin, ring finger protein 19A gene was up-regulated in JQ1 stimulated BMDMs at 1 h and that the DEAD box polypeptide 39B, histone cluster 1, H1c, DIS3 mitotic control homolog-like 2 genes were up-regulated at 4 h (Additional file 1: Supplementary Tables S3 and S4). Based on a literature review, these genes do not have a well-established role in inflammation. Additionally, we confirmed with a DAVID Bioinformatics Resources gene ontology (GO) analysis (FDR 0.05) that JQ1 up-regulated transcripts were associated with cellular macromolecular complex assemblies and primary metabolic processes (Additional file 1: Supplementary Figure S3). Interestingly, Banerjee *et al.*^26^ reported that JQ1 produced a potent up-regulation of chromatin modification genes, including Sirt1, Hdac6, and multiple lysine demethylases (KDMs), as well as Hexim-1 in J-Lat 10.6 cells, which have a potential role for HIV reactivation. In our RNA-seq data, we could not identify any chromatin modification genes that were induced by JQ1 in BMDMs. Nevertheless, whether these genes have any functional role in JQ1-mediated modulation of macrophage activation will require further study.

### Functional and pathway analyses following JQ1 treatment in LPS-stimulated BMDMs

**Figure 4:**
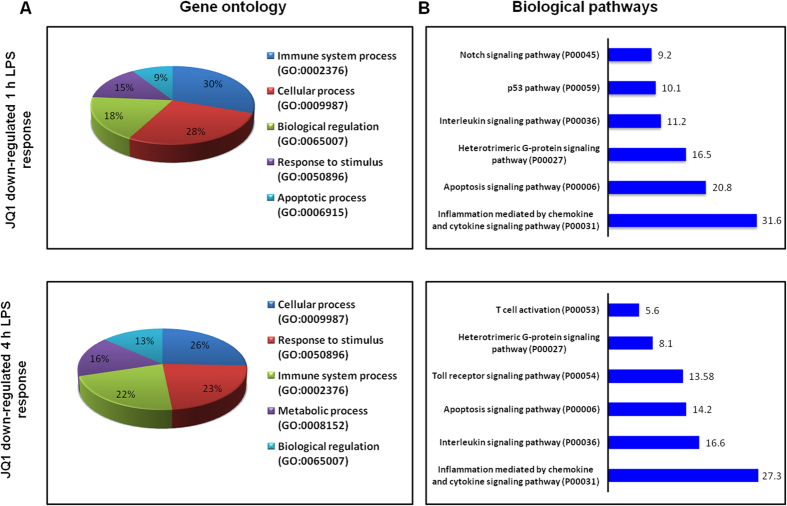
Functional annotation and biological pathways of the JQ1-down-regulated genes. (**A**) GO term enrichment analysis for the “biological process” category of the JQ1-down-regulated genes. The top GO terms are ranked by the count numbers. (**B**) The most highly represented biological pathways of the JQ1-down-regulated genes in the BMDMs.

The groups of LPS-up-regulated genes that showed a change in expression (*P* ≤ 0.01, and fold change ≥1.5 log_2_) were subjected to a GO analysis (FDR 0.05) with functional annotations using the DAVID Bioinformatics Resources and KEGG (Kyoto Encyclopedia of Genes and Genomes) pathways. DAVID revealed that all major biological processes and molecular functions within the GO analysis for the LPS up-regulated transcripts were, for the most part, genes associated with immune system processes, response to stimulus and biological regulation (Fig. 1F,G). To further functionally classify the JQ1-down-regulated genes (*P* ≤ 0.01, and fold change ≥1.5) with LPS stimulation, we again used the DAVID Bioinformatics Resources. Interestingly, we observed that the largest gene groups were involved in the same biological processes, e.g., immune system processes, response to stimulus and biological regulation (Fig. 4A). To determine the possible biological pathways of the JQ1-down-regulated genes (*P* ≤ 0.01, and fold change ≥1.5) in the LPS-treated BMDMs, we utilized the PANTHER classification system, version 9.0. The major categories of the biological pathways were inflammation mediated by chemokines and cytokines, apoptosis and interleukin signalling pathways (Fig. 4B).

### Confirmation of the differentially expressed genes by q-RT-PCR

**Figure 5:**
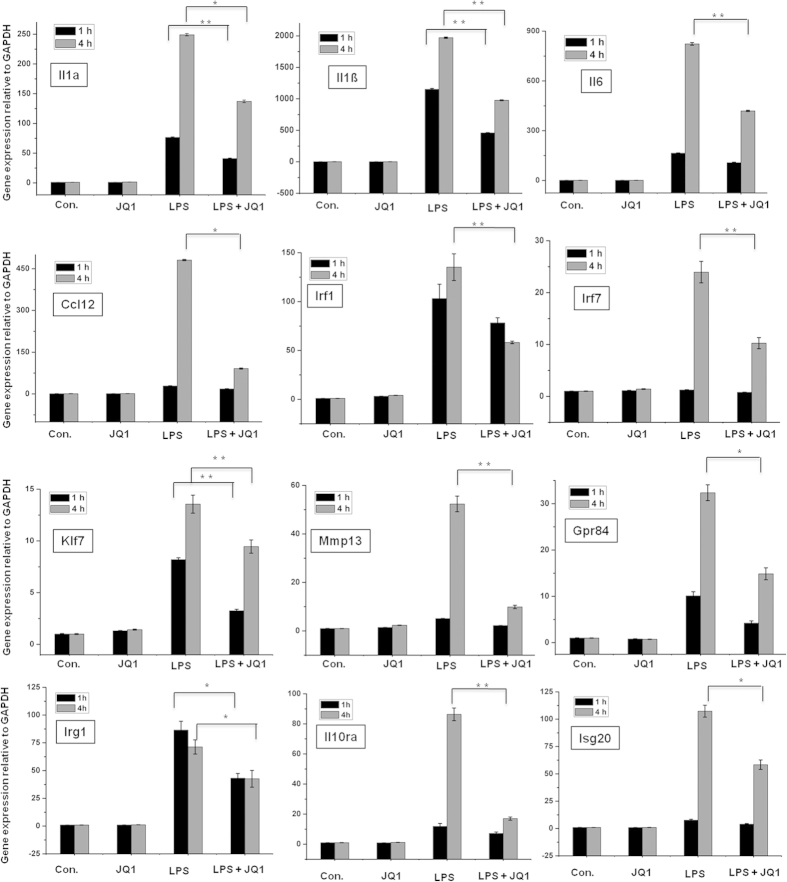
Confirmation of differentially expressed genes by quantitative reverse transcription-polymerase chain reaction. The Il1a, Il1ß, Il6, Ccl12, Irf1, Irf7, Klf7, Mmp13, Gpr84, Irg1, Il10ra, and Isg20 genes were significantly down-regulated in the JQ1-treated BMDMs. Gene expression was normalized to the GAPDH transcript levels. *P *<* 0.05 and **P *<* 0.001 compared with the control. The data represent three independent experiments.

**Figure 6:**
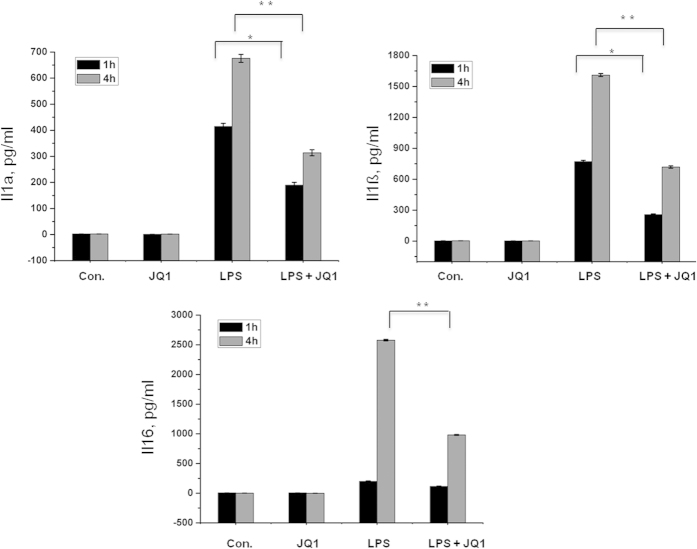
The BET family bromodomain inhibitor, JQ1 reduces LPS induced release of pro-inflammatory mediators. BMDMs supernatants from LPS and/or JQ1 co-treated cells were subjected to ELISA to detect the pro-inflammatory cytokine/chemokine levels. Therefore, primary microglial cells were treated with LPS and/or JQ1 for 1 and 4 h, followed by quantification of the Il1a, Il1ß, and Il6 levels. Values are given in pg/ml. Means and standard deviations of the mean of three independent experiments are shown (*P value *<* 0.05, **P value *<* 0.001).

A large number of genes that were identified as differentially regulated by the RNA-seq analysis were subjected to validation using real-time qRT-PCR, with GAPDH as the reference gene. Most were selected for validation according to the distinct effects of JQ1 on the LPS-affected genes. To measure gene expression, mRNA was reverse transcribed into cDNA using the Prime Script TM Reverse Transcriptase (Takara Bio Inc., Shiga, Japan) and the qRT-PCR assays were repeated several times using at least 3 mRNA preparations from independent experiments. The results are expressed as the fold change relative to the control levels. Thirteen genes were selected for verification, and the RNA-seq expression pattern was confirmed for nine genes (Il1a, Il1ß. Il6, Ccl12, Irf1, Irf7, Klf7, Mmp13, Gpr84, Irg1, Il10ra, and Isg20; Fig. 5); however, one was non-significant (data not shown) in the qRT-PCR analysis compared with the RNA-seq experiments. Additionally, we analysed cytokines/chemokines in the supernatants of treated primary microglial cells with ELISAs. Compared with untreated cells, the IL1a, IL1ß, and IL6 levels in the supernatants were increased in the BMDMs following 1 and 4 h of LPS treatment. Co-treatment with JQ1 led to significant reduction in the Il1a, Il1ß, and Il6 levels in the BMDMs (Fig. 6).

## Discussion

There are several small-molecule BET inhibitors that target diverse BET family members in cancer and inflammatory diseases. For example, a pan-BET inhibitor, I-BET, was proven to protect against LPS-induced endotoxic shock^14^. Another BET inhibitor disrupted the T-cell-mediated inflammatory response^28^. Among these inhibitors, JQ1 has attracted the most attention because of its significant efficiency in haematological malignancies^29^. Recently, other studies reported even wider prospective applications for JQ1, such as in attenuating lung fibrosis^30^, endotoxaemic shock^13^, NO synthesis and innate immunity^31^, suggesting that JQ1 may have anti-inflammatory activity. However, none of these studies addressed the effects of JQ1 at the genome-wide expression level in BMDMs. We examined BMDMs as a model of inflammation. This is one of the major experimental uses of primary macrophages. For the first time, in the present study, we showed an anti-inflammatory effect for JQ1 on genome-wide mRNA level changes in BMDMs, which is a model system for studying inflammation, using RNA-seq analysis. This unbiased profiling approach revealed that the importance of BET proteins in the regulation of key inflammatory genes involved in the establishment of innate immunity in BMDMs.

The results show that the stimulation of BMDMs with LPS up-regulated numerous inflammatory genes, including Nos2, II12b, II2rg, II1β, II1a, II6, II27, II15ra, II15, II18, II10ra, II13ra1, II1rn, Tnf-α, Ptgs2, Cxcl1, Cxcl2, Cxcl3, Cxcl9, Cxcl10, Cxcl16, Cx3cl1, Ccl5, Ccl22, Ccl4, Ccl7, Ccl2, Ccrl2 Ccl3, Ccl12, and Ccl9. Treatment of BMDMs with JQ1 resulted in the down-regulation of 38 and 203 (*P* ≤ 0.01, and fold change ≥1.5) LPS-inducible genes at 1 h and 4 h, respectively. Additionally, it suppressed key LPS-inducible inflammatory genes, including II1β, II1a, II6, II15ra, II18, II18bp, II10ra, II13ra1 II1rn, Irg1, Ccl2, Ccl5, Cxcl3, Cxcl9, Cx3cl1, Ccl12, and Ccl22, as well as the TFs, Irf7, Irf8, Irf9, and klf7 (Figs. 3A–E). The II1 family is one of the most studied pro-inflammatory gene families, and the most extensively studied forms regarding inflammatory disorders are Il1a, Il1β, and Il1 receptor antagonist (Il1rn) genes^32^. Il1 expression plays a crucial role in the pathogenesis of several diseases, including rheumatoid arthritis, inflammatory bowel disease, cardiovascular disease, chronic periodontitis, and osteoporosis^33^. Il1β plays a central role in inflammatory and immune processes, and Il1β activity blockade has entered clinical medicine as a therapy^34^. Il6 is a circulating cytokine that is known to be released from a number of different cells, including activated macrophages, and lymphocytes. Additionally, an elevated Il6 level is an important hallmark of rheumatoid arthritis and Crohn’s disease^35^,^36^. Thus, the down-regulation of the Il1 family genes (Il1a, Il1β, Il1rn) and Il6 through JQ1 could inhibit inflammatory disorders in BMDMs.

Our RNA-seq data revealed that JQ1 treatment inhibited the expression of important chemokines in LPS-activated BMDMs, for example, Ccl2, Ccl5, Ccl12, Ccl22, Cxcl3, Cxcl9, and Cx3cl1 (Figs. 3C,D). These chemokines, also referred to as inflammatory cytokines, and their excessive production has been associated with disease progression and severe inflammation pathologies^37^. Ccl2 and Ccl5 are potent chemoattractants for monocytes/macrophages and are highly expressed in rheumatoid arthritis patient synovial fluid and sera^38^,^39^. Additionally, Gosling J *et al.*^40^ reported that Ccl2 deletion attenuated diet-induced atherosclerosis in mice. Cx3cl1/Fractalkine is chemotactic for monocytes and lymphocytes and also serves as a cellular adhesion molecule. In rheumatoid arthritis, macrophages, fibroblasts, endothelia and dendritic cells expressed high Cx3cl1 levels. Furthermore, Cx3cl1 expression was observed after post-adjuvant injection in rat induced arthritis disease^41^. These effects are in agreement with reports showing that JQ1 can modulate the functional activities of immune cells and exert immunosuppressive effects by inhibiting cytokine and chemokine production.

One of the most striking features is that JQ1 significantly suppressed the expression of previously unidentified inflammatory genes that are induced by LPS in BMDMs. Mmp13 plays a pivotal role in arthritis pathogenesis and was found in the synovial tissue from patients with osteoarthritis or arthritis^42^. Recently, Singh A, *et al.*^43^ reported that the deletion of Mmp13 attenuated serum-induced arthritis. Thus, drugs that inhibit Mmp13 expression may be possible therapeutic agents for osteoarthritis or arthritis diseases. MDL-1—a C-type lectin domain family 5, member A (Clec5a)—is highly expressed on Tnf-α activated macrophages^44^, and during joint inflammation activation, Clec5a enhances myeloid cell infiltration and promotes Il1, Il6, Il17a, and Tnf-α expression, resulting in severe cartilage damage and bone erosion^45^. Orphan G protein-coupled receptor 84 (Gpr84), which is highly expressed in leukocytes, monocytes, and macrophages upon activation by LPS, plays a critical role in immunological regulation^46^. In the present study, JQ1 also significantly inhibited Mmp13, Clec5a, and Gpr84 expression (Fig. 3E–G), and these inhibitory effects of JQ1 may play a potential role in immune disorder treatment, possibly through inhibition of macrophages and the ensuing inflammatory responses. However, the mechanism by which JQ1 inhibits key inflammatory genes requires further study. Interestingly, it was demonstrated that BET proteins, i.e., Brd2, are essential for proinflammatory cytokine production in macrophages and that Brd2, as well as Brd4, physically associates with inflammatory cytokine gene promoters in macrophages. JQ1 evacuation of Brd4 from specific gene promoters leads to anti-inflammatory and anti-osteoclastogenetic effects, without ruling out that some effects of JQ1 are derived from inhibiting other members of the BET family^14^. Nevertheless, further investigations are needed to determine the selective roles of each BET protein (Brd2, Brd3, Brd2 and Brdt) in inflammatory gene regulation.

Another hallmark of inflammation is the increased expression of TFs, such as Irf7, Irf8 and Irf9. Here, we show that JQ1 down-regulates the expression of LPS-inducible TFs. IRFs are a family of transcription factors that are involved in inflammatory diseases^47^. Irf7 and Irf8 are important regulatory factors in the pathophysiology of autoimmune diseases, including systemic sclerosis^48^, and in particular, increased Irf7 transcriptional activity has been associated with autoimmune disorders, including systemic lupus erythaematosus^22^. However, Irf9 is an important regulator in type 1 Irf signalling, which regulates antiviral responses^49^. Additionally, Irf8-knockout dendritic cells fail to produce Tnf-α and Il6 proinflammatory cytokines upon stimulation with TLR9 agonists^50^. Furthermore, JQ1 also inhibited the expression of a wide group of other interferon-stimulated genes (ISGs), for example, Ifi205, Oas2, Oas3, Ifit3, Ifi47, Isg20, Zbp1, and Ifih1 in LPS-stimulated BMDMs (Figs. 3C,D). Previously, Nicodeme *et al.*^14^ reported that another BET protein inhibitor, I-BET, suppressed the expression of Irf4 and Irf8, but not Irf7 and Irf9, in BMDMs. In the present study, we showed that JQ1 down-regulated the expression of Irf7, Irf8 and Irf9 and their target genes in BMDMs. Thus, the down-regulation of Irf7, Irf8 and Irf9 through JQ1 could inhibit inflammatory disorders. Finally, the results achieved by the real-time RT-PCR analysis of Il1a, Il1ß. Il6, Ccl12, Irf1, Irf7, Klf7, Mmp13, and Gpr84 (Fig. 5) illustrate an essential down-regulation in the expression of the above-mentioned mRNAs in JQ1-treated BMDMs when compared with controls.

In the absence of LPS stimulation, the treatment of BMDMs with JQ1 had a marginal effect on gene transcription and did not have an impact on inflammatory gene expression (Figs 3C,D). Thus, the impact of JQ1 on LPS-inducible gene expression is highly selective. Most interestingly, crucial inflammatory genes, including Tnf-α and Nfκbia (Additional file 1: Supplementary Figure S2), as well as other inflammatory and immunity-related genes, such as Ptgs2, Nos2, Ccl3, Ccl4, Cxcl1, and Cxcl2, were unaffected by JQ1. This specificity and anti-inflammatory potential of JQ1 was validated using qRT-PCR analysis (data not shown). In our study, we observed that the prominent transcription factors Irf7, Irf8, Irf9, and Klf7 were suppressed by JQ1, although surprisingly, JQ1 had no effect on the master transcription factors NF-κB or AP-1. Therefore, it seems likely that the LPS-induced induction of Tnf-α, Tnfaip2, and Ccl3 transcription depends on NF-κB or AP-1 rather than the Irf7, Irf8, Irf9, and Klf7 transcriptional pathways^51^,^52^. This is an important area that we are actively pursuing further.

Overall, the RNA-seq genome-wide analysis that was utilized in this study identified LPS-inducible genes that were significantly suppressed or unaffected by JQ1, providing a clue for the selective effect of JQ1 on gene expression. However, further extensive *in vivo* experimentation studies will be required to investigate the anti-inflammatory effects of JQ1 and the mechanisms by which JQ1 inhibits key inflammatory genes, which will ultimately result in the development of effective and safe anti-inflammatory drugs.

## Conclusion

In summary, this study focused on the anti-inflammatory potential of the synthetic compound, JQ1. Our RNA-seq data revealed for the first time the gene expression changes that are induced by JQ1 in an inflammatory cell model, BMDMs, targeting inflammatory diseases. The findings suggest that JQ1 selectively inhibits the expression of several immune- and inflammation-related genes, including cytokines, chemokines, interleukins and interferons, to exert its anti-inflammatory function, and that JQ1 could be a candidate for inflammatory disease prevention.

## Methods

### Isolation, culture, and stimulation of BM-derived macrophages

C57BL/6 mice were purchased from Samtako Bio Korea (Gyeonggi-do, Korea), and the mice were maintained under specific-pathogen-free conditions. BMDMs were isolated from C57BL/6 mice as previously described^53^. All of the experimental protocols were conducted in accordance with our Institutional Animal Care and Use Committee (IACUC) guidelines and were approved by the Hanyang University IACUC committee (HY-IACUC-2014-0119). To initiate differentiation, the medium was supplemented with 25 ng/ml recombinant macrophage colony-stimulating factor (R&D Systems 416-ML) for 4 days. BMDMs were grown in Dulbecco’s Modified Eagle’s Medium (DMEM; Life Technologies, Carlsbad, CA, USA) that was supplemented with 10% foetal bovine serum (FBS) and 4 mM glutamine (Life Technologies, Carlsbad, CA, USA). The cells were maintained in a humidified incubator with a 95% air, 5% CO_2_ atmosphere at 37 °C. JQ1 was purchased from Cayman Chemicals (Ann Arbor, MI, USA) and dissolved in dimethyl sulfoxide (DMSO, Sigma-Aldrich) as a 10 mM stock solution; the stock solution was diluted in DMEM for the experiments. The final DMSO concentration in the medium was less than 10 μL/10 mL, which did not produce any cell growth effects. The cells were simultaneously treated with a well-tolerated concentration, i.e., 1 μM, of JQ1 along with 100 ng/mL LPS (Sigma Aldrich) and incubated for 1 and 4 h under normal culture conditions.

### Total RNA extraction

Total RNA (~8 μg) was extracted using TRIzol® (Life Technologies, Carlsbad, CA, USA) according to the manufacturer’s instructions. Briefly, two hundred microliters of chloroform was added to tubes containing the lysis mixture, which were then inverted gently for 5 minutes (min). The mixture was centrifuged at 12,000 x *g* for 15 min at 4 °C, and the clear upper solution was placed into a new tube, to which 500 μl isopropanol was added. The tubes were inverted before they were incubated on ice for 1 h. The lysis mixture was centrifuged at 12,000 x *g* for 10 min at 4 °C, and the isopropanol was decanted. Ice-cold 70% ethanol was added to the RNA pellet for gentle washing. After centrifuging as above for 10 min, the ethanol was removed. The RNA pellets were dried at room temperature for 5–10 minutes before reconstitution in 20 ml RNase-free water, and the RNA was treated with RNase-free DNase (Promega, Wisconsin, USA). The RNA quality was assessed using an Agilent 2100 Bioanalyzer with the RNA 6000 Nano Chip (Agilent Technologies, Waldbronn, Germany), and the quantity was determined using a spectrophotometer (NanoDrop Technologies, Wilmington, DE, USA).

### Quantitative real-time RT-PCR

Reverse transcription of the RNA samples was performed as described^54^ using 2 μg of total RNA, 1 μl random hexamers (per reaction) and the Prime Script 1st-strand cDNA synthesis kit (Takara, Japan). The random hexamers and RNA templates were mixed and denatured at 65 °C for 5 min., followed by cooling for 2 min on ice. Prime Script buffer (5x), RTase and RNAse inhibitor were added to the cooled template mixture and incubated for 1 h at 50 °C before enzyme inactivation at 70 °C for 15 min. qRT-PCR was performed using the SYBR Green PCR Master Mix (Takara Bio Inc., Shiga, Japan) and a 7500 fast real-time PCR system (Applied Biosystems, Foster City, USA). Glyceraldehyde-3-phosphate dehydrogenase (GAPDH) was used as an internal control. Complementary DNA samples were diluted 1.5-fold, and qRT-PCR was performed using an AB-7500 Real-time thermal cycler (Applied Biosystems, Foster City, USA) with SYBR Premix Ex-Taq II (Takara Bio, Shiga, Japan) according to the manufacturer’s directions. The reactions were produced in a 20-μl volume with 0.4 mM of each primer (Table 1). Each PCR run included a no-template control with water instead of cDNA and a reverse transcriptase-negative control for each gene. Triplicate measurements were performed for all reactions. Different samples were evaluated using 96-well plates for the gene expression experiments, and all of the samples were analysed on a single plate for endogenous control determinations. The results were analysed using the critical threshold (∆C_T_) and comparative critical threshold (∆∆C_T_) methods in the AB-7500 software with the Norm finder and the geNorm-plus algorithms. The primers were designed using Primer Express (Applied Biosystems, Foster City, USA).

### cDNA library preparation for RNA-Seq

Total RNA was extracted from sixteen independent BMDM samples, i.e., control 1 h (2 samples), control 4 h (2 samples), JQ1 1 h (2 samples), JQ1 4 h (2 samples), LPS 1 h (2 samples), LPS 4 h (2 samples), LPS+JQ1 1 h (2 samples) and LPS+JQ1 4 h (2 samples), using TRIzol® (Life Technologies, Carlsbad, CA, USA) according to the manufacturer’s protocol. For the RNA-seq, RNA libraries were created from each group using the NEBNext® Ultra™ Directional RNA Library preparation kit from Illumina®. The first step in the workflow involved the removal of ribosomal RNA using the RNAMius™ Transcriptome Isolation kit (Life Technologies, Carlsbad, CA, USA). Following purification, total RNA was fragmented into small pieces using divalent cations at an elevated temperature. The cleaved RNA fragments were copied into first-strand cDNA using reverse transcriptase and random primers, followed by second-strand cDNA synthesis using DNA polymerase I and RNase H. The cDNA fragments were then processed through an end-repair reaction by the addition of a single ‘A’ base, followed by ligation of the adapters. The products of these reactions were then purified and enriched using PCR to create the final cDNA library. The cDNA fragments were sequenced using the Illumina HiSeq 2500 (101 cycles PE lane) (National Instrumentation Center for Environmental Management at Seoul National University). Biological replicates (n = 2) were utilized for each BMDM condition in the RNA sequencing experiments.

### Differential gene expression analysis

Raw sequence files underwent a quality control analysis using FastQC (version 0.10.1, http://www.bioinformatics.babraham.ac.uk/projects/fastqc/). To avoid low-quality data, we clipped and trimmed the reads using the FASTX-Toolkit (version 0.0.14, http://hannonlab.cshl.edu/fastx_toolkit/). For the analysis of differentially expressed genes, the quality-checked read data for each condition were processed using the TopHat (version 2.0.10)^55^ software, based on a reference genome sequence (*Mus musculus* UCSC mm10), and the differential gene expressed values of each sample were calculated using Cufflinks^56^, based on the “fragments per kilobase per million map reads” (FPKM) method^25^. The data from each condition were combined separately, which produced eight biological datasets, and the genes whose expression levels significantly differed were identified. We used a 1% false discovery rate (FDR), *P* ≤ 0.01, and fold change ≥1.5 log_2_ for up- or down-regulation as the criteria for defining differentially expressed genes. The RNA-seq experiments were visualized using HOMER (version 4.7) after preparing custom tracks for the UCSC Genome Browser (http://genome.ucsc.edu/). The acquired data were deposited in the sequence read archive (http://www.ncbi.nlm.nih.gov/sra/) under the dataset accession numbers SRR1825641, SRR1825642, SRR1825643, SRR1825644, SRR1825646, SRR1825647, SRR1825649, SRR1825650, SRR1825654, SRR1825655, SRR1825656, SRR1825657, SRR1825658, SRR1825659, SRR1825660, and SRR1825661.

### Functional annotation and pathways

The DAVID (Database for Annotation, Visualization and Integrated Discovery) (version 6.7) software (http://david.abcc.ncifcrf.gov/home.jsp) was used to determine the most functional annotations of the significant genes in the datasets, as described previously^57^,^58^. DAVID calculates a modified Fisher’s exact P value to demonstrate gene ontology (GO) or molecular pathway enrichment. Values less than 0.05 were considered to be strongly enriched in the annotation category. To determine the possible biological pathways involved in the JQ1-treated BMDMs, a gene classification analysis of the down-regulated genes was performed using the PANTHER classification system version 9.0 (http://www.pantherdb.org), as described previously^59^. Genes from the datasets that were associated with biological pathways in the PANTHER Pathways Knowledge Base were considered for literature analysis.

### Ingenuity Pathway Analysis for TFs identified target genes

IPA (Ingenuity Systems, http://www.ingenuity.com, Mountain View, CA, USA) was conducted to analyse the TF predicted target genes in response to LPS stimulation as previously described^24^,^54^,^60^. The genes from the datasets that were associated with canonical pathways in the Ingenuity Pathways Knowledge Base (IPAKB) were considered for the identified TF predicted target genes analysis. Fisher’s exact test *P* values indicated the probability that an association could be explained by chance. After uploading the datasets, TF identifiers were mapped to corresponding gene objects.

### Enzyme-linked immunosorbent assay (ELISA)

BMDMs were cultured in the same condition as above and treated with LPS, JQ1, and LPS + JQ1 for one and four hours. After treatment, the concentration of the pro-inflammatory mediators, I Il1a, Il1ß, and Il6 were determined in cell culture supernatants using mouse ELISA kits (Koma Biotech, Seoul, Korea) according to the manufacturer’s protocol.

### Statistical analysis

The data were analysed using Origin Pro 8 (Origin Lab Corporation, Northampton, MA, USA). Each value is expressed as the mean ± standard error of the mean (SEM). The statistical analyses were performed using SPSS 17.0 (SPSS Inc., IL, USA). The data were tested using a one-way ANOVA, followed by the Tukey’s HSD post hoc test. ^*^P < *0.05* and ^**^P < *0.001* were considered significant.

## Additional Information

**How to cite this article**: Das, A. *et al.* Dual transcriptome sequencing reveals resistance of TLR4 ligand-activated bone marrow-derived macrophages to inflammation mediated by the BET inhibitor JQ1. *Sci. Rep.* **5**, 16932; doi: 10.1038/srep16932 (2015).

## Supplementary Material

Supplementary Information

## Figures and Tables

**Table 1:** List of primers used in qRT-PCR studies.

Gene designation	Forward (5′ ->3′)	Reverse (5′ ->3′)
*Il1a*	TCTATGATGCAAGCTATGGCTCA	CGGCTCTCCTTGAAGGTGA
*Il1b*	GAA ATG CCA CCT TTT GAC AGT G	CTG GAT GCT CTC ATC AGG ACA
*Il6*	TAG TCC TTC CTA CCC CAA TTT CC	TTG GTC CTT AGC CAC TCC TTC
*Ccl12*	ATTTCCACACTTCTATGCCTCCT	ATCCAGTATGGTCCTGAAGATCA
*Irf1*	ATG CCA ATC ACT CGA ATG CG	TTG TAT CGG CCT GTG TGA ATG
*Irf7*	GCGTACCCTGGAAGCATTTC	GCACAGCGGAAGTTGGTCT
*Klf7*	AGT GGA CAT TTT GCT CTC TCG	GTT AAT GAG GTC ACT GCG TTG A
*Mmp13*	GACAGTGGAGGTGGCCTTAC	GGTCTCAAAAGGGCGACTGA
*Gpr84*	TCTCATTGCTCTAGGACGCTAC	AGACAAAAACATTCCAGAGGGG
*Irg1*	GGCACAGAAGTGTTCCATAAAGT	GAGGCAGGGCTTCCGATAG
*Il10ra*	CCCATTCCTCGTCACGATCTC	TCAGACTGGTTTGGGATAGGTTT
*Isg20*	GAGGGCTGTTGGTTCTTGACT	CCTCGGGTCGGATGTACTTG
*Gapdh*	TGCGACTTCAACAGCAACTC	CTTGCTCAGTGTCCTTGCTG
