# Supplementary material for: Dual transcriptome sequencing reveals resistance of TLR4 ligand-activated bone marrow-derived macrophages to inflammation mediated by the BET inhibitor JQ1
Source: Sci Rep. 2015 Nov 19;5:16932. doi: 10.1038/srep16932 (PMC4652239; doi:10.1038/srep16932)
Supplement: Supplementary Information [file srep16932-s1.pdf]

Title: Dual transcriptome sequencing reveals resistance of TLR4 ligand-activated bone marrow-derived macrophages to inflammation mediated by the BET inhibitor JQ1.

Amitabh Das [a], Jin Choul Chai [b], Chul-su Yang [b], Young Seek Lee [b], Nando Dulal Das [c], Kyoung Hwa Jung [d] **, Young Gyu Chai [a, b,] *

[a]*Department of Bionanotechnology, Hanyang University, Seoul, 133-791, Republic of Korea*

[b]*Department of Molecular & Life Sciences, Hanyang University, Ansan, 426-791, Republic of Korea*

[c]*Epigenetics Drug Discovery Unit, Division of Structural & Synthetic Biology, RIKEN Center for Life Science Technologies, 1-7-22 Suehiro-cho, Yokohama 230-0045, Japan*

[d]*Institute of Natural Science & Technology, Hanyang University, Ansan, 426-791, Republic of Korea*

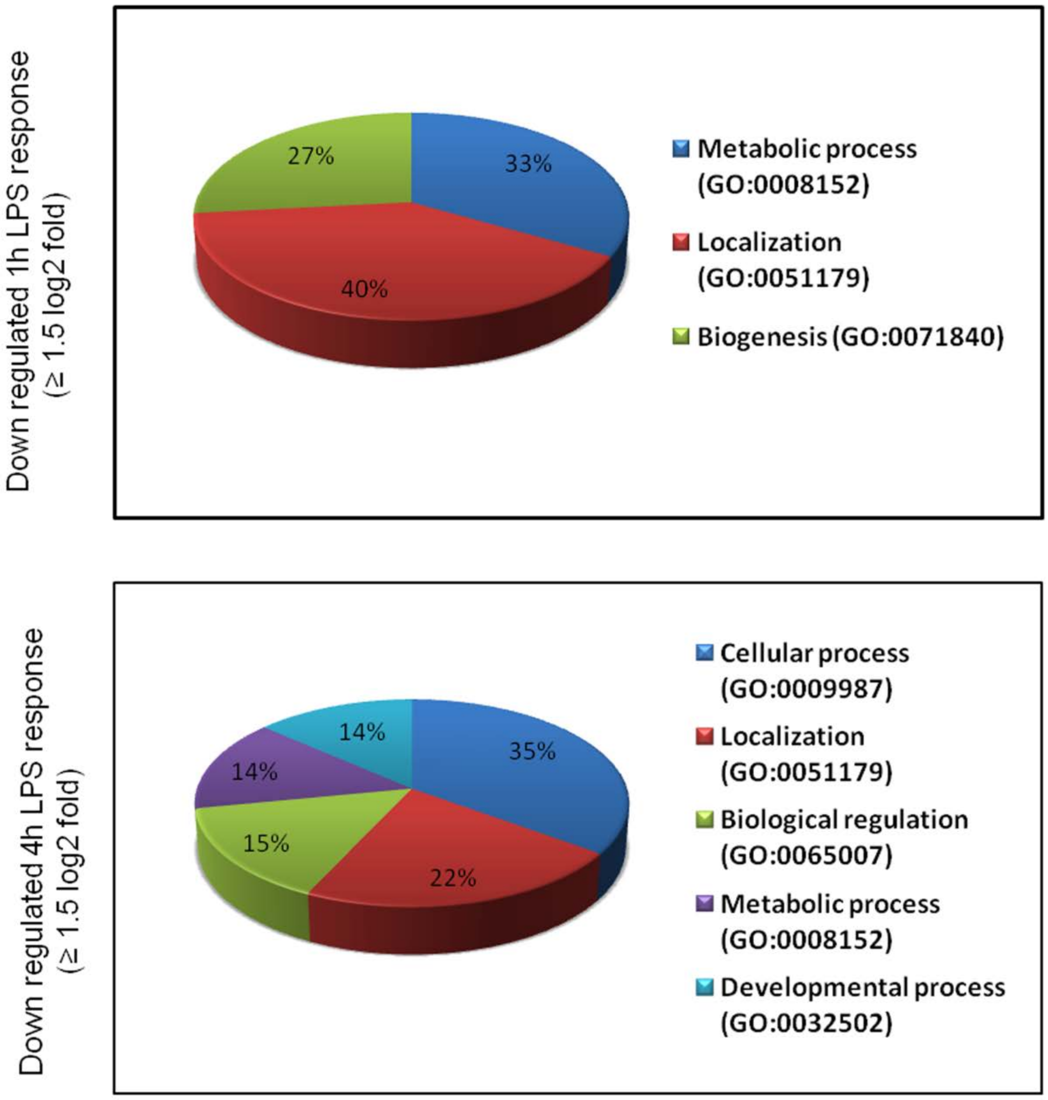

Figure S1 Functional annotations of LPS-inducible down-regulated genes. Gene Ontology analysis of functional annotations (biological process) associated with 1 and 4 h LPS-inducible down-regulated genes in BMDMs comparison with the control.

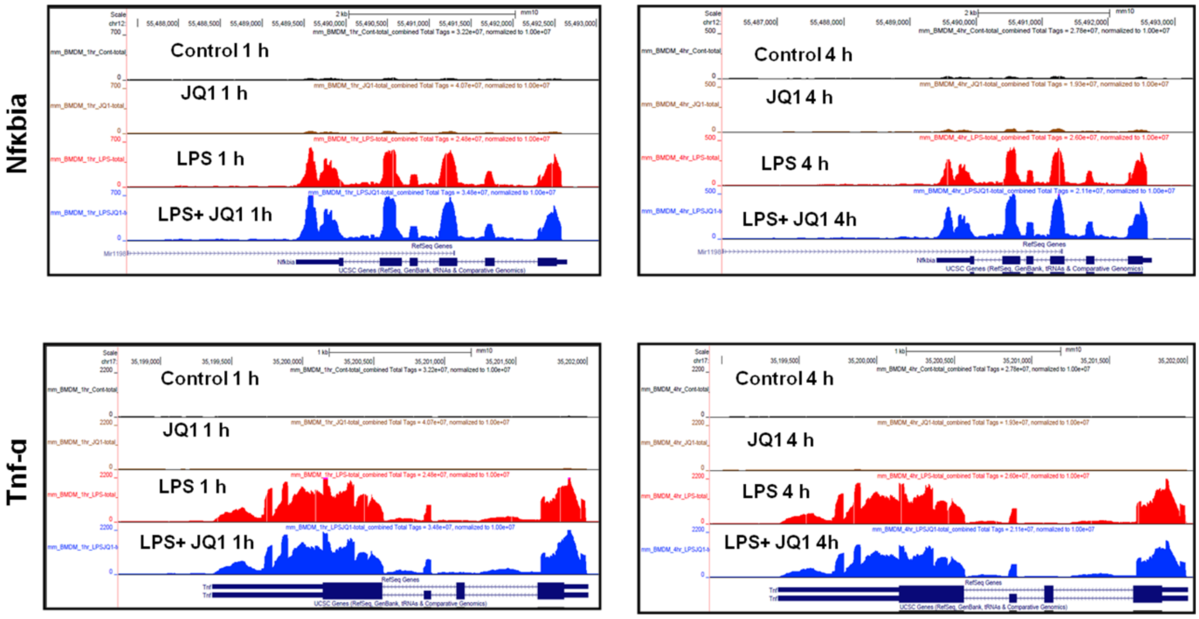

Figure S2 JQ1 did not affect a specific subset of LPS-inducible genes. UCSC Browser images representing the normalized RNA-seq read densities of inflammatory genes that were un-affected by JQ1 after 1 and 4 h in LPS-stimulated BMDMs compared with the control.

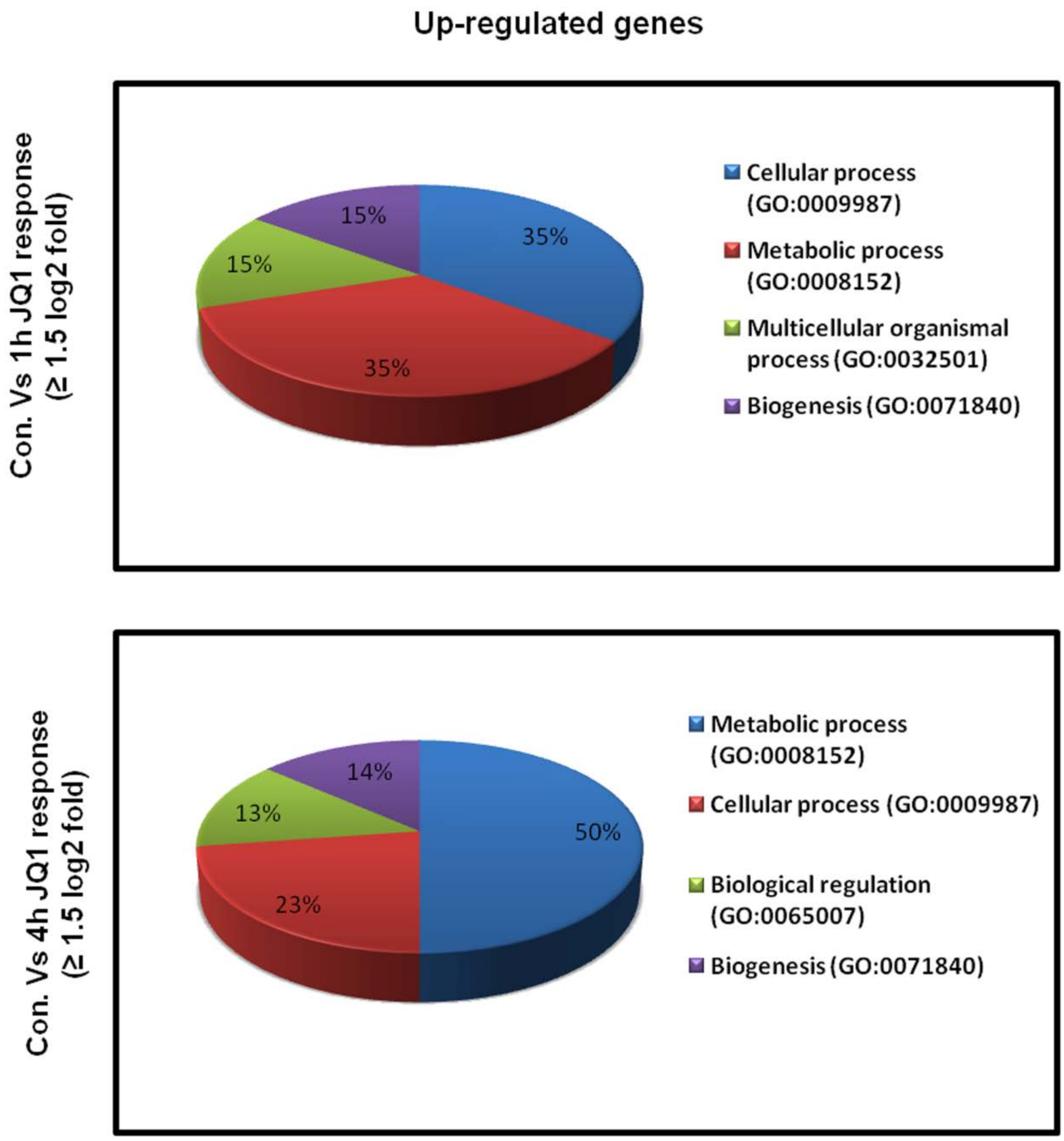

Figure S3 Functional annotation of JQ1-inducible genes. Gene ontology analysis of functional annotations (biological process) that were associated with 1 and 4 h JQ1-inducible up-regulated genes in BMDMs, in comparison with the control.

Table S1: Top 20 significant down-regulated genes in 1 h LPS stimulated BMDM

Gene Accession_ID	Gene Symbol	Expression log_2 (fold_change)	*P*-Value
NM_026597	Fam212a	-3.53367	0.0141
NM_001029990	Mettl17	-3.28127	0.0108
NM_033079	M1ap	-2.89804	0.0037
NM_001004362	2610008E11Rik	-2.88758	0.00005
NM_145530	Rhov	-2.79232	0.008
NM_001042591	Arrdc3	-2.18721	0.00005
NM_020581	Angptl4	-2.18594	0.0015
NM_001039106	Ddhd1	-2.16849	0.0008
NM_026734	Tmem126b	-2.08056	0.0014
NM_138313	Bmf	-2.05618	0.00005
NM_025573	Srsf9	-2.05218	0.010
NM_145713	Hist1h1d	-1.95017	0.002
NM_013843	Zfp53	-1.95017	0.00005
NM_153063	Zfp472	-1.9119	0.00085
NM_001190302	Gm14326	-1.80295	0.0001
NM_001009935	Txnip	-1.77072	0.00005
NM_175116	Lpar6	-1.69503	0.00005
NM_175662	Hist2h2ac	-1.68198	0.00005
NM_025537	Tsfm	-1.6661	0.00224
NM_001166497	3110052M02Rik	-1.56736	0.00025

Table S2: Top 20 significant down-regulated genes in 4 h LPS stimulated BMDM

Gene Accession_ID	Gene Symbol	Expression log_2 (fold_change)	*P*-Value
NM_026436	Tmem86a	-5.81357	0.012
NM_199221	Cd300lb	-5.413	0.00005
NM_001038703	Gpr146	-5.23545	0.0024
NM_144538	Rab3il1	-5.06741	0.0028
NM_001163495	Arhgap19	-4.78714	0.00005
NM_178901	AI467606	-4.66306	0.0028
NM_001036740	B3gnt8	-4.64243	0.00225
NM_008425	Kcnj2	-4.34056	0.00005
NM_001013370	Sesn1	-4.24005	0.001
NM_145509	5430435G22Rik	-4.23618	0.00005
NM_172589	Lhfpl2	-4.07019	0.00005
NM_008856	Prkch	-4.04472	0.00005
NM_009911	Cxcr4	-4.03993	0.00005
NM_007678	Cebpa	-4.01451	0.00005
NM_001146180	Mtss1	-3.92722	0.00005
NM_008494	Lfng	-3.85228	0.00005
NM_008535	Lyl1	-3.82364	0.00005
NM_007901	S1pr1	-3.80906	0.00005
NM_001271411	Nfam1	-3.80632	0.00005
NM_145554	Ldlrap1	-3.79495	0.00005

Table S3: Significant up-regulated genes in 1 h JQ1 stimulated BMDM

Gene Accession_ID	Gene Symbol	Expression $\log_2$ (fold_change)	*P*-Value
NM_175542	Rttn	4.9905	0.00005
NM_013923	Rnf19a	3.9153	0.0004
NM_016981	Slc9a1	3.6439	0.00005
NM_001012309	Ccdc55	3.5240	0.002
NM_177089	Tacc1	2.7312	0.00005
NM_013650	S100a8	1.7571	0.003

Table S4: Significant up-regulated genes in 4 h JQ1 stimulated BMDM

Gene Accession_ID	Gene Symbol	Expression $\log_2$ (fold_change)	*P*-Value
NM_001252457	Ddx39b	1.9217	0.00005
NM_015786	Hist1h1c	1.7614	0.00005
NM_001172157	Dis3l2	1.5013	0.007

Additional file 2: Different TFs predicted to be activated top 400 transcript in LPS 4h response

Stat1 predicted to be activated (p=2.07 e-66)		Nfkbia predicted to be activated (p=4.05 e-35)		Irf1 predicted to be activated (p=4.42 e-38)	
ID	Fold Change	ID	Fold Change	ID	Fold Change
Usp18	7.249	Traf1	6.569	Trim21	4.035
Trafd1	2.767	Tnfsf10	6.628	Tnfsf10	6.628
Tnfsf10	6.628	Tnfaip3	4.996	Tnf	6.953
Tnf	6.953	Tnfaip2	4.139	Tlr3	3.878
Tgtp2	7.104	Socs3	7.236	Tap2	3.337
Tap1	4.217	Slfn2	3.765	Tap1	4.217
Sp110	3.821	Saa3	8.168	Socs7	2.781
Socs3	7.236	Ptgs2	11.676	Rsad2	8.603
Slfn5	4.813	Nos2	9.960	Ptgs2	11.676
Slfn2	3.765	Lcn2	4.636	Pml	3.324
Slfn8	4.353	Jdp2	3.719	Oas1g	3.705
Slfn4	7.555	Itgav	2.658	Nos2	9.960
Samhd1	2.991	Isg15	7.642	Jak2	4.627
Rsad2	8.603	Il6	10.050	Isg15	7.642
Ptgs2	11.676	Il1rn	4.202	Il27	8.828
Psme2	2.816	Il1a	10.275	Il1b	10.276
Oasl1	8.725	Il15	5.282	Il18	4.217
Nos2	9.960	Ifit1	8.086	Il15	5.282
Mx1	7.982	Ifi205	8.946	Il12b	10.889
Lcn2	4.636	Icam1	5.920	I830012O16Rik	6.820
Jak2	4.627	H2-Q7	4.639	Ifit2	7.787
Isg15	7.642	Gch1	4.335	Gm14446	5.649
Irgm1	4.915	Gbp2	7.288	Ifih1	5.525
Il6	10.050	Gadd45b	3.701	Ifi35	3.870
Il15ra	6.536	Cxcl1	9.241	Gbp2	7.288
Il15	5.282	Cx3cl1	8.960	Fpr2	5.334
Igtp	5.397	Cp	5.017	Fgl2	4.184
I830012O16Rik	6.820	Cflar	4.531	Eif2ak2	3.820

Ifit2	7.787	Cd86	3.401	Cxcl10	10.955
Ifit1	8.086	Ccl7	8.255	Cdkn1a	5.037
Gm14446	5.649	Ccl5	9.739	Cd40	7.868
Ifi47	6.226	Ccl3	6.025	Cd274	4.776
Ifi35	3.870	Ccl2	6.409	Ccl5	9.739
Ifi205	8.946	Casp4	3.627	Il6	10.050
Icam1	5.920	Tnf	6.953	Ccl12	8.206
Herc6	5.146	Sdc4	4.923	Cfb	5.993
Gbp6	7.247	Nod2	5.319	Oas2	4.267
Gbp5	10.184	Mmp14	4.606	Psme2	2.816
Gbp2	7.288	Mmp13	5.644		
Fgl2	4.184	Il1b	10.276		
Fam26f	5.855	Il15ra	6.536		
Eif2ak2	3.820	Fscn1	6.179		
Edn1	7.063	Cxcl2	10.672		
Cxcl9	11.185	Cxcl10	10.955		
Cxcl10	10.955	Cdkn1a	5.037		
Cx3cl1	8.960	Ccl12	8.206		
Cmpk2	8.130	Birc3	3.601		
Cdkn1a	5.037	Bcl2a1a	9.034		
Cd86	3.401	C3	4.970		
Cd40	7.868	Cd40	7.868		
Cd274	4.776	Cd69	9.494		
Ccrl2	6.111	Csf1	6.081		
Ccl5	9.739	Pim1	4.534		
Ccl12	8.206	Sod2	4.772		
Casp4	3.627				
C3	4.970				
Batf2	6.134				
Pdgfrb	6.356				
Il12b	10.889				

Fcgr2b	2.709
Cxcl2	10.672
Ccl4	6.346
Ccl3	6.025
Ccl2	6.409
Cfb	5.993
Il1b	10.276
Oas2	4.267
Pim1	4.534
Tlr3	3.878
